# Evading the strength–ductility trade-off dilemma in steel through gradient hierarchical nanotwins

**DOI:** 10.1038/ncomms4580

**Published:** 2014-04-01

**Authors:** Yujie Wei, Yongqiang Li, Lianchun Zhu, Yao Liu, Xianqi Lei, Gang Wang, Yanxin Wu, Zhenli Mi, Jiabin Liu, Hongtao Wang, Huajian Gao

**Affiliations:** 1 LNM, Institute of Mechanics, Chinese Academy of Sciences, Beijing 100190, China; 2 Laboratory for Microstructures, Shanghai University, Shanghai 200444, China; 3 National Engineering Research Center for Advanced Rolling Technology, University of Science and Technology Beijing, Beijing 100083, China; 4 Institute of Applied Mechanics, Zhejiang University, Hangzhou, Zhejiang 310027, China; 5 School of Engineering, Brown University, Providence, Rhode Island 02912, USA

## Abstract

The strength–ductility trade-off has been a long-standing dilemma in materials science. This has limited the potential of many structural materials, steels in particular. Here we report a way of enhancing the strength of twinning-induced plasticity steel at no ductility trade-off. After applying torsion to cylindrical twinning-induced plasticity steel samples to generate a gradient nanotwinned structure along the radial direction, we find that the yielding strength of the material can be doubled at no reduction in ductility. It is shown that this evasion of strength–ductility trade-off is due to the formation of a gradient hierarchical nanotwinned structure during pre-torsion and subsequent tensile deformation. A series of finite element simulations based on crystal plasticity are performed to understand why the gradient twin structure can cause strengthening and ductility retention, and how sequential torsion and tension lead to the observed hierarchical nanotwinned structure through activation of different twinning systems.

## Abstract (web summary)

It is a long-standing goal in metallurgy to enhance the strength of materials without sacrificing ductility. Here, the authors demonstrate that applying pre-torsion to a twinning-induced plasticity steel generates a hierarchical nanotwin structure, improving strength whilst retaining ductility.

## Graphical abstract

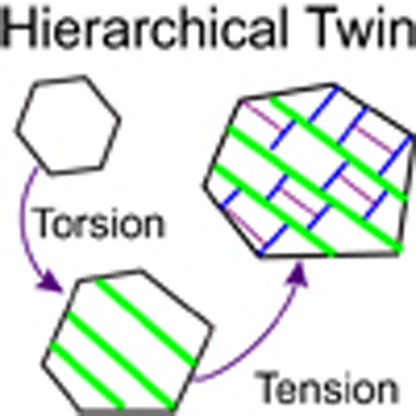

For the essential role played by steels in infrastructural and overall economic development^1^,^2^, modern steelmakers are heavily involved in the development of new advanced steels, by tuning their compositions or by adopting new processing routines, to meet the increasing demands for high-performance materials^2^,^3^,^4^. In this field, sustained effort for innovation has led to the development of high manganese twinning-induced plasticity (TWIP) steels with exceptional combinations of formability, hardenability and ultimate strength, properties that are often attributed to the prominence of twinning-induced deformation in these materials^3^,^4^,^5^. First discovered in 1888 by Sir Robert Hadfield^6^, TWIP steels have attracted significant attention over the last decade^7^ due to the compelling needs of weight reduction and improved crash safety in modern vehicle components^8^. As shown in Supplementary Fig. 1, while the ultimate strength of TWIP steels can reach 1.4–1.6 GPa, their yielding strength is as low as ~300 MPa^7^. Given the general correlation between yielding strength and fatigue limit^9^, an important challenge is to increase the yielding strength of TWIP steels without compromising ductility. Unfortunately, traditional material processing techniques for strength enhancement, such as grain refinement^10^,^11^,^12^,^13^ or cold working, usually result in reduced ductility^14^. Supplementary Fig. 1 shows that strength and ductility are mutually exclusive in steels, based on data from US Steel Cooperation^15^, which reflects the long-standing dilemma of strength–ductility trade-off in materials science^10^.

Significant developments have been achieved in recent years in processing metals to optimize their properties. Examples include introducing twins as second-level structures in grains^16^,^17^,^18^, generating relatively sharp grain size gradient^19^,^20^, or producing hierarchical structures with nanoscale grains and sub-granular solutes^21^, as well as gradient grains with embedded twins^22^,^23^. While these studies have substantially advanced the fundamental science of nanostructured materials^24^,^25^,^26^,^27^, it remains an open question how to scale them up to bulk engineering materials for broad industrial applications.

Inspired by the ideas of introducing nanotwin structures^16^,^17^,^18^ and grain size gradient to enhance both ductility and strength of metals^19^,^20^, here we report a method to enhance the strength of TWIP steel by introducing a linearly graded nanotwinned structure in the material, with no trade-off in ductility and no limitations on sample dimensions. The latter will be essential in enabling practical applications of the method developed to enhance any axially symmetric structural components, including axles in machines, engines and transmission systems in mechanical, civil, aerospace, transportation, oil, automotive and energy industries. For example, with rapid population growth and the resulting demand for high-speed rail transport over much of the world in the coming century, the axles in high-speed trains pose critical safety concerns where high strength, ductility and fatigue life will be particularly desired.

## Results

### Mechanical behaviour of pre-torsioned TWIP steel

**Figure 1:**
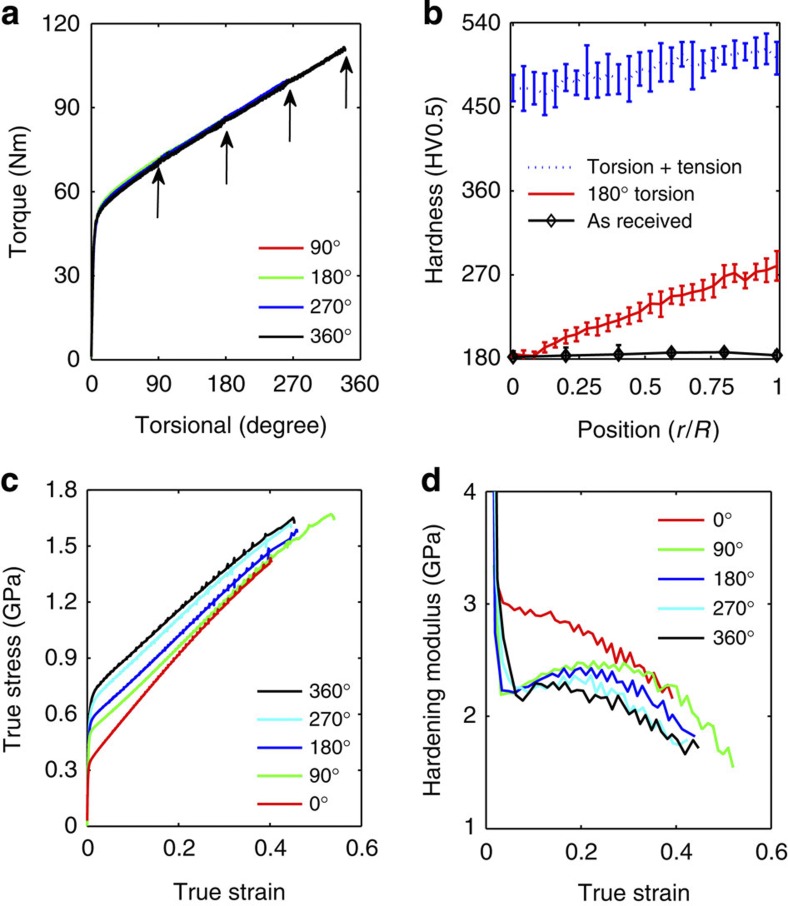
Strengthening of FeMnC TWIP steel by pre-torsion treatment. (**a**) Torsion experiments on samples at four different torsional angles. The resultant shear strain *γ* is a function of position *r* along the radial direction, *γ*=*θrL*^−1^, where *θ* is the overall twist in a selected region with uniform diameter and length *L*=70 mm. For a nominal pre-torsion of 180°, the maximum shear strain in the sample with *R*=5 mm is ~0.2, where *R* is the radius of the sample. (**b**) Variation of hardness in the radial direction. Values represent average from *n*=10 tests (error bars: s.d.). Data are for as-received samples, samples subject to 180° torsion, and those subject to 180° pre-torsion and subsequent tension. (**c**) Stress–strain curves for the pre-torsioned samples. (**d**) Hardening modulus as a function of strain upon stretching of pre-torsioned samples.

The FeMnC TWIP steel is used in our study. The detailed processing in making such material is given in the Methods section. A low stacking fault energy in the range of 15–30 m Jm^−2^ in the austenitic structure^4^ is realized, which favors the formation of mechanical twins^3^,^5^,^7^. Such TWIP steel has a typical yielding strength of ~300 MPa but superior hardening capacity, resulting in increased uniform elongation and high ultimate strength^3^,^5^,^7^,^8^. We first apply torsion to a TWIP steel bar with dimensions shown in Supplementary Fig. 2a. The torque versus twist curve given in Fig. 1a shows not only the repeatability of experiments but also enormous hardening in the material. Typical shape of a sample with pre-torsion is given in Supplementary Fig. 2b. We first measure the variation of micro hardness of the sample along the radial direction. The hardness is clearly seen to increase along the radial direction in the sample with pre-torsion (see Fig. 1b). The stress–strain behaviours shown in Fig. 1c indicate that pre-torsion has led to substantial increases in yielding strength and also slight enhancements in ultimate strength in the tested TWIP steel samples. Supplementary Fig. 1 shows that the simultaneous attainment of both strength and ductility in FeMnC TWIP steel with pre-torsion is in striking contrast to the usual strength–ductility trade-off in steels. The hardenability, as quantified by hardening modulus, is superior for all samples subject to pre-torsion and remains at a high value till sample failure, as shown in Fig. 1d. The serrations in the stress–strain responses in TWIP steel are likely due to reorientation of some crystals during twin deformation^5^, which may have also resulted in the observed fluctuations in the hardening behaviour, as seen in Fig. 1d. All of our TWIP samples failed in shear mode, with and without pre-torsion, as seen in Supplementary Figs 2c and d.

### Microstructure evolution and deformation mechanisms

**Figure 2:**
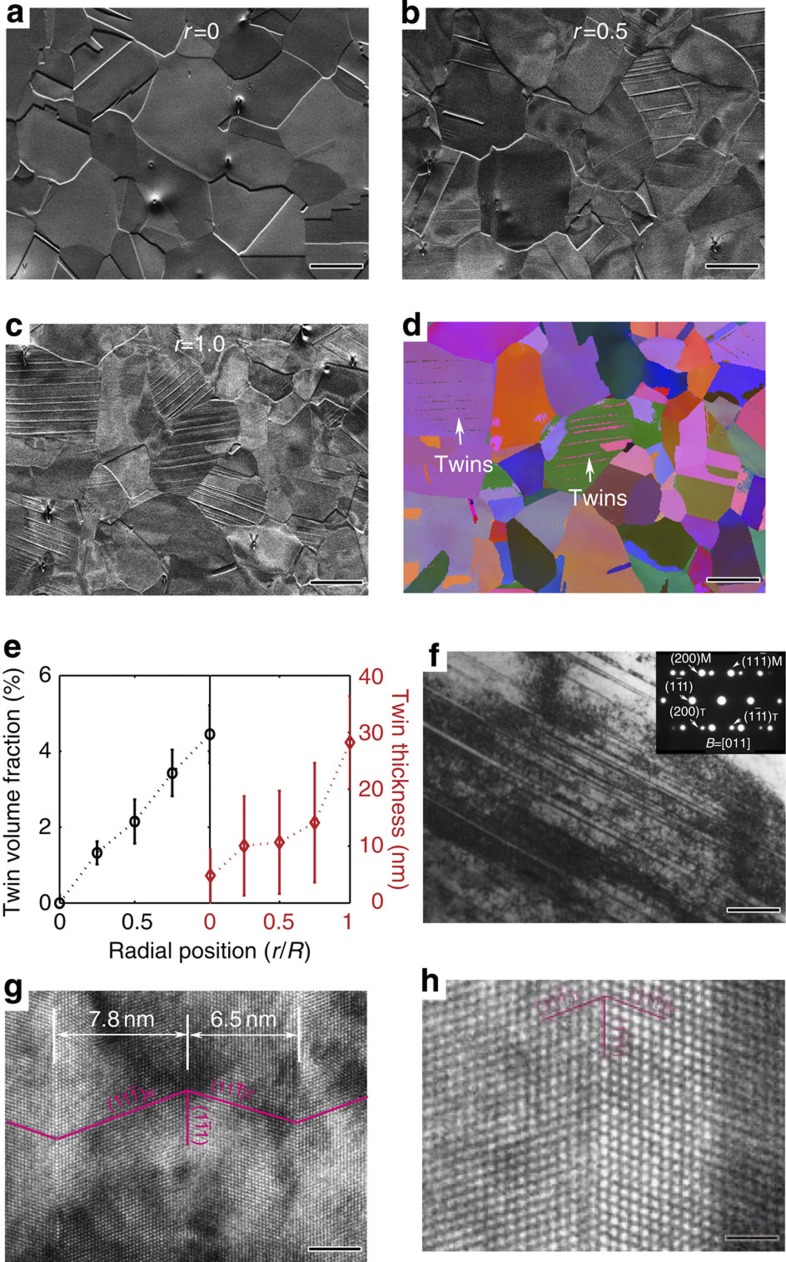
Microstructures showing a gradient nanotwin structure along the radial direction of a 180° pre-torsioned TWIP sample. (**a**–**c**) SEM images of microstructures at different radial positions, *r*/*R*=0, 0.5, 1, respectively. The as-rolled TWIP steel has a grain size of ~32 μm (scale bars 30 μm). (**d**) EBSD image of microstructure in (**c**) to confirm that the band structures are twin laminae (scale bar, 30 μm). (**e**) Evolution of twin volume fraction and twin thickness as functions of the radial position in the pre-torsion sample (error bar for twin volume due to measurements covering a surface area of ~0.1 mm^2^; error bar for twin thickness from the s.d. of 30 data points). (**f**) TEM image of twins at *r*/*R*=1.0. The inset is the selected area electron diffraction pattern of the twins (scale bar, 0.3 μm). (**g**) Typical nanoscale twin structure (scale bar, 3 nm). (**h**) High-resolution TEM image of a clean twin boundary introduced by pre-torsion (scale bar, 1 nm).

**Figure 3:**
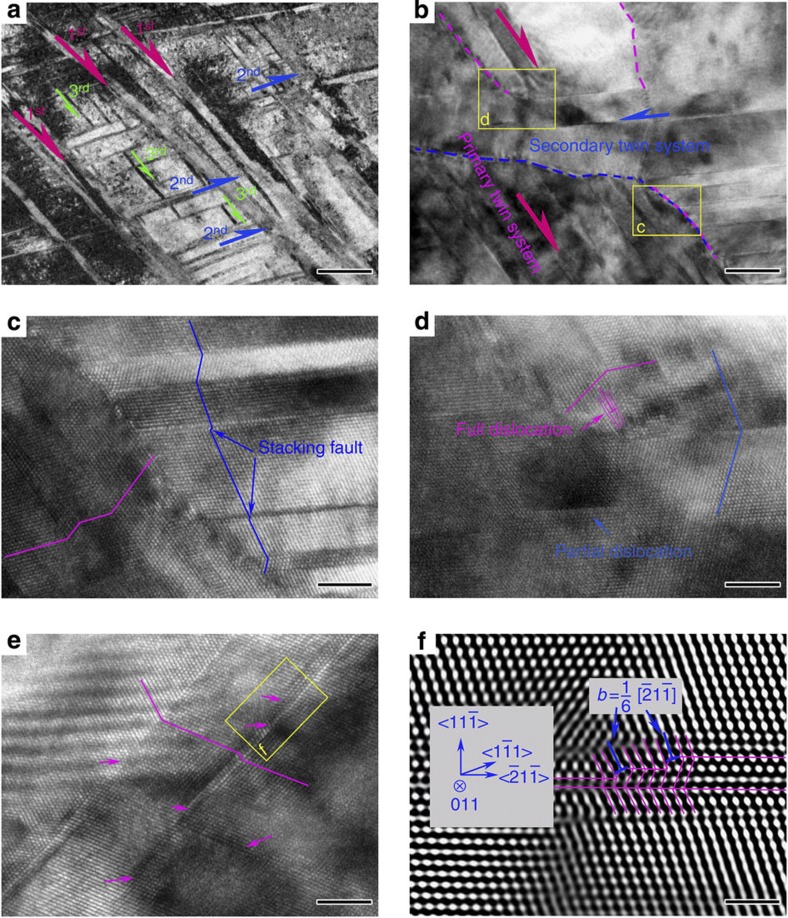
Hierarchical twin structures and interactions between dislocations and deformation twins. Atomic scale details near the outermost region of the 180° pre-torsioned sample after tensile failure are presented, and interactions between dislocations and deformation twins with pre-existing twin boundaries are examined. (**a**) Primary twins running from top left to bottom right (pink arrows), secondary twins in inclined orientations (blue arrows) and short tertiary twins between secondary twins parallel to the primary ones (green arrows) (scale bar, 0.3 μm). (**b**) HRTEM image showing the formation of twin junctions when secondary twins penetrate the primary twins (scale bar, 9 nm). (**c**) The enlarged image of the yellow rectangle ‘c’ in (**b**), showing the lattice arrangement of primary twins and secondary twins (scale bar, 3 nm). (**d**) Close view of the yellow rectangle ‘d’ in (**b**), showing both full dislocations and partial dislocations near the junction (scale bar, 3 nm). (**e**) Plenty of partial dislocations on the twin boundaries (scale bar, 3 nm). (**f**) The corresponding inverse fast Fourier transformation image of (**e**) to identify the dislocations in the yellow rectangle in (**e**) as 
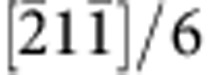
 Shockley partials (scale bar, 1 nm).

To understand the mechanism of plastic deformation in TWIP samples with pre-torsion, we found it particularly insightful to examine the variations in twin density along the radial direction. Figure 2a–c presents microstructures in the sample with 180° pre-torsion at different radial positions, *r*/*R*=0, 0.5, 1, respectively. The scanning electron microscope (SEM) images reveal a gradient twin structure: the pre-torsion resulted in mechanical twins whose density increases with radial distance from the centre, with the outermost region (*r*/*R*=1) of the sample displaying the highest twin density. Figure 2d shows an electron backscatter diffraction (EBSD) image of Fig. 2c, which confirms that the band structures in Fig. 2c are indeed twin laminae. Complementary EBSD images to SEM pictures shown in Fig. 2a–c are also given in Supplementary Fig. 3. At increasing radial distance from the centre, not only the twin density increases but the twins also thicken, as seen in Fig. 2e. Figure 2f,g provides transmission electron microscope (TEM) images of twin boundaries at *r*/*R*=1.0 introduced during pre-torsion, where typical twin thickness is on the order of tens of nanometres. The twin boundaries are in general very clean, as seen in the high-resolution TEM image in Fig. 2h. Another important feature, as shown in Fig. 2a–d, is that twins in each grain are parallel and are identified as primary twins at the stage of pre-torsion. The finely spaced parallel twins can serve as barriers to inclined twinning or dislocation slip and/or source for further twinning, leading to the distinct hardening behaviours between samples with or without pre-torsion, as evidently seen in Fig. 1d. The secondary twins are very scarce right after torsion. However, upon subsequent stretching to failure under tension, the pre-torsioned samples exhibit a hierarchical nanotwinned structure with abundant secondary twins and even tertiary twins in the outermost region, as shown in Fig. 3.

Figure 3a shows primary twins running from top left to bottom right (pink arrows), secondary twins in inclined orientations (blue arrows) and short tertiary twins between secondary twins parallel to the primary ones (green arrows). Secondary twins are constrained by primary twins and tertiary twins are confined between the secondary ones. Hence, the primary twins serve as effective barriers for the secondary twins, and the latter renders formation of the tertiary twins harder. Both mechanisms contribute to strain hardening during plastic deformation. Primary and secondary twins are also confirmed in the corresponding selected area electron diffraction spots with <011>-beam incident (Supplementary Fig. 4). In addition to twin–twin interaction, activities between twin boundaries and dislocations are of particular interests for strength hardening and ductility. A dislocation approaching a twin boundary may dissociate, with one partial passing through the boundary and gliding in a conjugate slip system, and the other residing and moving along the twin boundary^18^,^28^. Combining with experimental observations, a thorough examination of possible reactions between dislocations and twin boundaries in f.c.c. metals is also available^29^. Figure 3b shows an HRTEM image on the formation of twin junctions when secondary twins penetrate the primary twins. Figure 3c provides an enlarged image of the yellow rectangle ‘c’ in Fig. 3b with detailed lattice arrangement of primary and secondary twins. A close view of the yellow rectangle ‘d’ in Fig. 3b, showing full and partial dislocations near the junction interface, is given in Fig. 3d. Plenty of partial dislocations are observed on the twin boundaries, as can be seen in Fig. 3e and the corresponding inverse fast Fourier transformation image (Fig. 3f). Dislocations in the yellow rectangle in Fig. 3e are identified as 
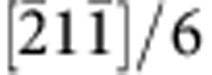
 Shockley partials. The dissociation of such dislocations when interacting with twin boundaries avoids excessive stress concentration due to dislocation pile-up allows the material to deform and hence plays a crucial role in retaining ductility.

### Influence of gradient and hardening on strength and ductility

The gradient twin density shown in Fig. 2 in TWIP steels with pre-torsion and the hierarchical nanotwinned structure after further tensile deformation in those twisted samples seen in Fig. 3 suggest the significance of twin gradient for retaining ductility. To explain why the combination of gradients and twin structures are so beneficial for both strength and tensile ductility, we have performed a series of finite element simulations based on isotropic plasticity (Supplementary Note 1) and crystal plasticity models that account for all the operative slip and twinning systems in TWIP steel (Supplementary Note 2). Our simulations show that the yielding strength of a gradient sample obeys the law of mixture 
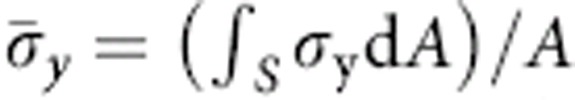
 where *A* denotes the area of shear plane *S*. This behaviour can be understood by noting that, since the sample is homogeneously deformed while yielding, any material point in the sample should be in its plastic regime.

**Figure 4:**
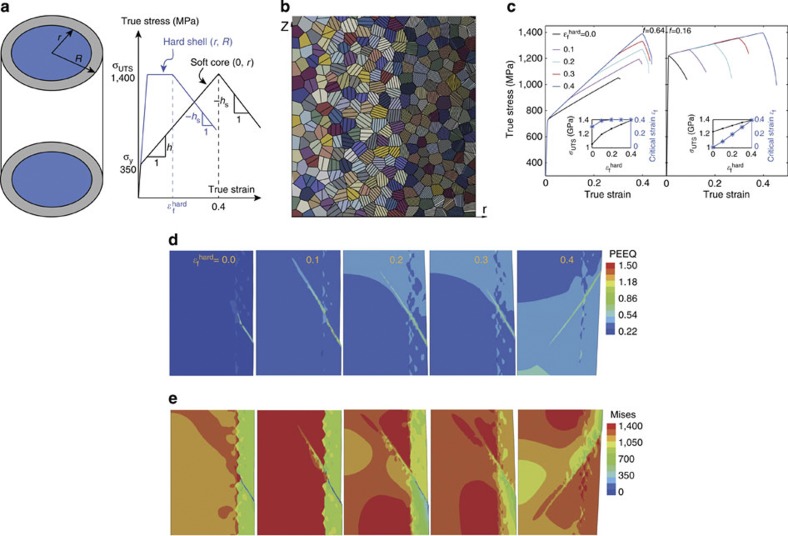
Finite element simulations showing the influence of the failure strain on shear localization of a gradient twin structure. (**a**) A cylindrical sample with a hard shell and soft core with distinct stress–strain behaviours. The same softening modulus *h*_s_=1.75 GPa is used for both core and shell. (**b**) A representative gradient microstructure composed of uniform voronoi grains with increasing twin density from the core to the surface of the cylindrical sample. Only one-third of the sample (along *z* axis) is shown for better view. (**c**) Stress–strain behaviours of the gradient twin sample for different values of the failure strain 
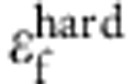
 in the hard shell, and different volume fractions of the soft core (*f*=*r*^2^*R*^−2^) *f*=0.64 (left) and *f*=0.16 (right). The inset for *f*=0.64 suggests that the failure strain *ε*_f_ of the gradient sample is not sensitive to 
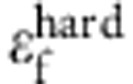
. When *f*=0.16, however, *ε*_f_ is very sensitive to 
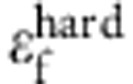
. (**d**) Equivalent plastic strain contours after the stress peak for different 
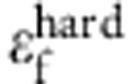
 in the sample with *f*=0.64, showing clearly shear localization. (**e**) Corresponding von Mises stress contours (in unit of MPa).

We first considered a cylindrical sample with a hard shell and soft core with distinct stress–strain behaviour (Fig. 4a). The volume fraction of the soft core is defined as *f*=*r*^2^*R*^−2^. Two volume fractions *f*=0.16 and *f*=0.64 of the soft core are considered. Using *f*=0.64 as an example, grains within the soft core region 0<*r*<0.8*R* have material properties described by the black stress–strain curve in Fig. 4a, while grains in the hard shell region from 0.8*R* to *R* have properties described by the blue curve. Figure 4b shows a representative two-dimensional (2D) polycrystalline microstructure used in the calculations. From the stress–strain behaviours of macroscopic samples with varying 
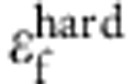
 in the hard shell material (Fig. 4c), it is observed that the yielding strength of all samples is ~730 MPa for *f*=0.64 and 1,230 MPa for *f*=0.16, which are dramatically higher than the yielding strength (350 MPa) of the soft core. These results are consistent with the simple law of mixture 
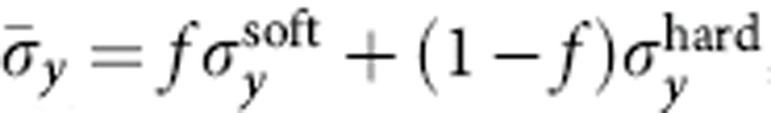
, where 
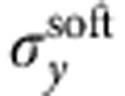
=350 MPa and 
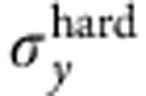
=1,400 MPa are the yielding strengths of the soft core and hard shell, respectively. More importantly, we observe that in the *f*=0.64 samples where the soft core dominates, the critical strain where the whole sample softens is very close to 0.4, the critical strain of the soft core. When the hard shell softens and the soft core still undergoes hardening, the average hardening modulus is 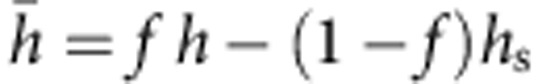. For *f*=0.64, 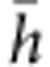 remains positive until the soft core starts to soften, which is why the critical strain could approach closely to 0.4. In contrast, for *f*=0.16, 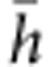 becomes negative as soon as the hard shell starts to soften, and the ductility is now controlled by the hard shell. These simulations demonstrate that the combination of non-hardenable strong shell with highly hardenable soft core could improve the strength while retaining the ductility of the soft core material, which is consistent with experimental observations^20^.

Tensile ductility is closely linked to mechanical instabilities. There exist two types of instabilities in tension: one leads to diffusive necking, with the critical point of maximum load described by the Considère criterion; the other creates localized necking and subsequent fracture. For the latter type, Hill^30^ and Rice^31^ developed general instability criteria for elastic–plastic solids by treating the localization of plastic deformation as a bifurcation from smoothly varying deformation into highly concentrated shear bands. Hardenability is the central parameter of the Hill–Rice instability criteria. In samples with gradient properties, an effective hardening modulus can be defined as 
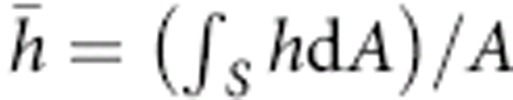
. Supplementary Fig. 5 and Supplementary Note 1 show our simulation results on the response of a core-shell gradient cylindrical sample with fixed mechanical properties for both the hard shell and soft core, but changing volume fraction *f* of the soft core. Supplementary Fig. 5b indicates that there exists an abrupt transition in failure strain *ε*_f_, the parameter quantifying ductility, at a critical value of *f.* The transition occurs at the point when 
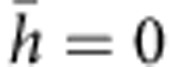
 and confirms the prediction by Rice^30^ for plastic materials with smooth yield locus. The simulation results in Supplementary Fig. 6 clarify the influence of softening moduli on the strength and ductility of a sample with linear strength gradient, which resembles our pre-torsioned samples. The results reveal that the absence of factors that lead to strong softening (that is, instabilities, fracture, and so on) could enhance both the strength and ductility of the sample.

Our finite element simulations convincingly demonstrate that (i) the gradient twin structure could profoundly increase the yielding strength with respect to the soft core; (ii) the sample could retain the same failure strain as the soft core when its volume fraction reaches a certain amount; and (iii) the localization of an axi-symmetric, gradient sample in tension occurs when 
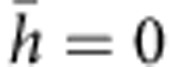
. We note that these conclusions are also applicable to materials with gradient in grain size.

Next we reveal another distinct deformation mechanism in gradient twin materials, in addition to the dislocation–twin interaction mechanisms discussed in Fig. 3, which is essential for retaining ductility in the pre-torsioned samples. In the simulations, we have assumed that pre-straining by torsion degrades the ductility of the sample (that is, the hard-core has higher yield strength but lower critical strain where softening starts); see Supplementary Fig. 6. This assumption could be conservative since deformation twinning by torsion in TWIP steel may not necessarily degrade the ductility. During subsequent tension, parallel twins after torsion (see Fig. 2) may give way to other twin systems prone to be activated. Comparison of pole figures of the pre-twisted samples before and after tension (Supplementary Fig. 7) indeed suggests the activation of different twinning systems by torsion and by tension.

### Activation of different twinning systems for high ductility

**Figure 5:**
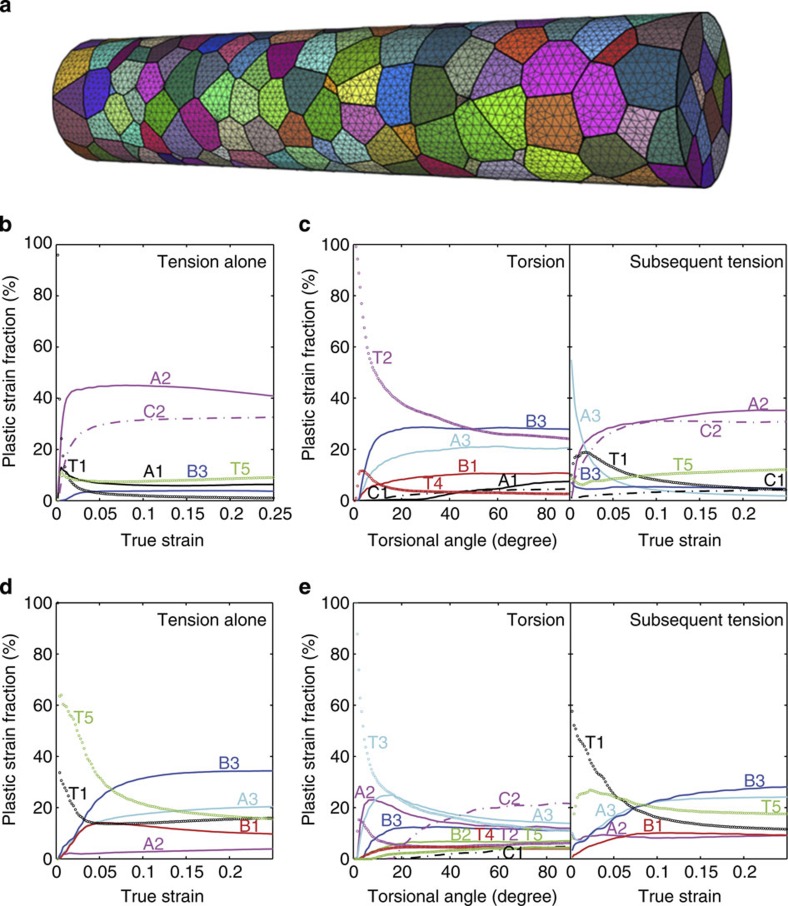
Crystal plasticity simulations showing a transition of twinning systems during torsion and subsequent tension. (**a**) The 3D polycrystalline microstructure with mesh adopted in the model. The plastic strains contributed by individual slip systems and twinning systems in grain one (G1, Euler angles (*ϕ*, *θ*, *ω*)=(17.5°, 178°, 172°)) and grain two (G2, (*ϕ*, *θ*, *ω*)=(52.5°, 53°, 137°)) near the surface under different loading conditions. (**b**) G1, tension; (**c**) G1, torsion and subsequent tension; (**d**) G2, tension; (**e**) G2, torsion and subsequent tension.

More precise calculations based on a crystal plasticity model that accounts for both dislocation slip and deformation twinning are performed (Supplementary Note 2 and ref. 32). We simulated the plastic deformation of a cylindrical sample composed of multiple single crystals (see Fig. 5a). Two different boundary value problems, similar to the experimental set-up, were considered. For the first case, we apply simple tension to the bar until ~40% strain. The plastic strains contributed by individual crystallographic slip systems and twinning systems in a grain near the surface (G1, Euler angles (*ϕ*, *θ*, *ω*)=(17.5°, 178°, 172°)) are shown in Fig. 5b. We notice the dominant role played by deformation twinning. The plastic strains contributed by individual crystallographic slip systems and twinning systems in another typical grain near the surface (G2, Euler angles (*ϕ*, *θ*, *ω*)=(52.5°, 53°, 137°)) are given in Fig. 5d. In another independent simulation, we twist the bar first and then apply uniaxial tension. Activated twinning/slip systems and evolution of strains contributed by those systems are shown in Fig. 5c,e for G1 and G2, respectively. For G1, twinning deformation is dominated by (T2, T4) and the activated slip systems are (B3, A3, B1 and A1), while during subsequent tension, activated twin systems are now (T5, T1) and activated slip systems are (A3, A2, C2, C1 and B3). For G2, the activated twinning systems during torsion and those in subsequent tension are also largely different. These results of our crystal plasticity simulations confirm the hypothesis that preferential twin systems during pre-torsion may give way to the activation of other twin systems during subsequent tension, which results in a fully three-dimensional (3D), hierarchical twin network with controlled twin sizes and stress concentration. This minimizes the chance of excessive grain boundary deformation that usually accounts for premature failure. These factors help maintain ductility in the pre-torsioned TWIP steel samples and lead to non-simple additive plastic deformation through sequential torsion and tension. Supplementary Fig. 8 shows the microstructure in the core of the 180° pre-torsioned sample (near *r*/*R*=0 hence without deformation twins induced by torsion) after tensile failure. The formation of primary twins is evident (Supplementary Fig. 8a), and the twins are ~10 nm in thickness (Supplementary Fig. 8b). Braid-like twins due to severe plastic deformation are also observed (Supplementary Figs 8c,d), which may contribute to the enormous hardenability of the materials as well.

## Discussion

The present study reveals that the strength of FeMnC TWIP steel can be substantially enhanced without sacrificing ductility, by introducing a gradient twin density with parallel twins in individual grains via pre-torsion. In subsequent tension, the formation of secondary twins and twin junctions where several secondary twins meet at a primary twin suggest a hierarchical twin deformation mechanism that leads to the observed strength enhancement with non-compromising ductility and strain hardening. The pre-torsion and subsequent tension activate different twinning systems and lead to simultaneous high-strength, high-ductility and high-strain hardening.

We note that a gradient in grain size (equivalent to yielding strength) can effectively increase the strength of materials when loaded along the direction perpendicular to the gradient direction. However, gradient alone is not always sufficient for retaining ductility. To confirm this, we have applied the same pre-torsion treatment to 6061 Aluminium alloy samples, as seen in Supplementary Fig. 9. Supplementary Fig. 9a shows the torque versus twist curves for pre-torsion of 180° and 360°, while Supplementary Fig. 9b confirms the presence of a gradient deformation field with increasing hardness along the radial direction in the pre-torsioned sample. However, due to the lack of a hierarchical nanotwin network, since deformation twinning is rare to occur in Al at room temperature due to its high stacking fault energy, the process resulted in enhanced strength at the cost of reduced ductility as pre-torsion is increased (Supplementary Fig. 9c), and the hardening modulus decreases with stretching, dropping to zero at critical strain levels that decrease substantially as pre-torsion is increased (Supplementary Fig. 9d).

Back to the essential role of gradient hierarchical nanotwins in the observed phenomenon of strength enhancement with no ductility trade-off in TWIP steel, our theoretical analysis and simulations show that, while the gradient structure could dramatically increase the yielding strength with respect to the soft core of the samples, the overall material hardening due to the hierarchical twin structures and the resulting distributed plasticity due to twin–twin and dislocation–twin interactions play the dominant role for retaining ductility. Given the general correlation between fatigue limit and yielding strength in most metals and metallic alloys^10^, the enhanced yielding strength in pre-torsioned FeMnC TWIP steel could result in much better reliability of structures composed of such material. Note that the essence of our method is to produce non-uniform shear strains in metals prone to deformation twinning, and twins in the pre-processing stage are so oriented that subsequent deformation would trigger different twin systems. Following this guideline, other methods may be developed to enhance material performance in metal manufacturing.

## Methods

### Material preparation

The FeMnC TWIP steel used in this study is composed of (by weight) 23.84% manganese, 0.61% carbon and balanced iron. The material was prepared by induction melting in argon atmosphere. The molten alloy was cast to a flat ingot, followed with rolling at an initial temperature of 1,050 °C and at a finishing temperature of 975 °C. The final hot rolled plate is 26.0 mm in thickness. The plates then undergo air cooling. After cooling, we used wire electrical discharge machining to cut dog-bone specimens (Supplementary Fig. 1) from rolled samples along the rolling direction for further characterization.

### Mechanical testing

Torsion and tensile experiments were conducted by using MTS model 809 axial/torsional testing system. The torsion tests were performed at an angular loading rate of 0.05° s^−1^, and the tensile strain rate is ~10^−4^ s^−1^. We measured the hardness along the radial direction by using an MH-6 micro hardness tester, and the indentation load is 500 g for TWIP steel and 200 g for 6061 Aluminium. The dwelling time is 15 s. At each position along the radial direction, we conducted 10 independent measurements.

### X-ray diffraction

Pole figures of the initial and deformed samples were obtained by X-ray diffraction technique by using Siemens D5000 diffractometer with copper-K radiation.

### SEM characterization and EBSD analysis

MIRA3 (LM) field emission SEM from TESCAN was employed for microstructure characterization. We used AZTEC from Oxford Instruments to perform EBSD analysis and obtain the information for deformation twinning.

### TEM sample fabricated with focused ion beam

Dual-beam-focused ion beam/scanning electron microscope (FIB/SEM) method was used to prepare the TEM specimen. After deformation, regions of interests were obtained using wire electrical discharge machining, and the specimen was mounted into a FEI 600i dual-beam FIB/SEM. The surface of the specimen was coated with platinum to prevent charging and to reduce ion beam damage. A dense beam of Ga+ was used to mill deep trenches in the regions strained at different values along the radial direction. A foil was then prepared in an orientation perpendicular to the radial direction by excavating on both sides of it with the ion beam at an acceleration voltage of 30 kV and the maximum current of 20 pA. Exhumation of the foil (10 × 5 × 0.1 μm) from the specimen surface using an *in situ* micromanipulator was carried out to transfer it to a copper grid. Platinum soldering was used to fix the foil on the copper grid. After mounting the foil on the copper grid, we ion mill the foil again to reduce its thickness down to 50 nm.

### TEM observation

TEM observation is carried out by using a JEM-2100 TEM operated at 200 kV. Images are recorded on a charge coupled device camera (2 k × 2 k, Gatan 831) with mode of binning two.

## Author contributions

Y. Wei and H.G. conceived the project, developed the idea for experiments and wrote the manuscript; Y. Li, X.L. and Y. Wei performed mechanical tests and texture analysis; L.Z., Y. Liu, and Y. Wei. performed the simulations; Y. Wu. and Z.M. synthesized FeMnC TWIP steel samples; G.W. prepared the TEM samples; and J.L., H.W. and Y. Wei performed the (high-resolution) TEM observations; all authors were involved in data analysis and commented on the manuscript.

## Additional information

**How to cite this article:** Wei, Y. *et al.* Evading the strength–ductility trade-off dilemma in steel through gradient hierarchical nanotwins. *Nat. Commun.* 5:3580 doi: 10.1038/ncomms4580 (2014).

## Supplementary Material

Supplementary Information Supplementary Figures 1-9, Supplementary Table 1, Supplementary Notes 1-2 and Supplementary References
