# Supplementary material for: Evading the strength–ductility trade-off dilemma in steel through gradient hierarchical nanotwins
Source: Nat Commun. 2014 Apr 1;5:3580. doi: 10.1038/ncomms4580 (PMC3988817; doi:10.1038/ncomms4580)
Supplement: Supplementary Information — Supplementary Figures 1-9, Supplementary Table 1, Supplementary Notes 1-2 and Supplementary References [file ncomms4580-s1.pdf]

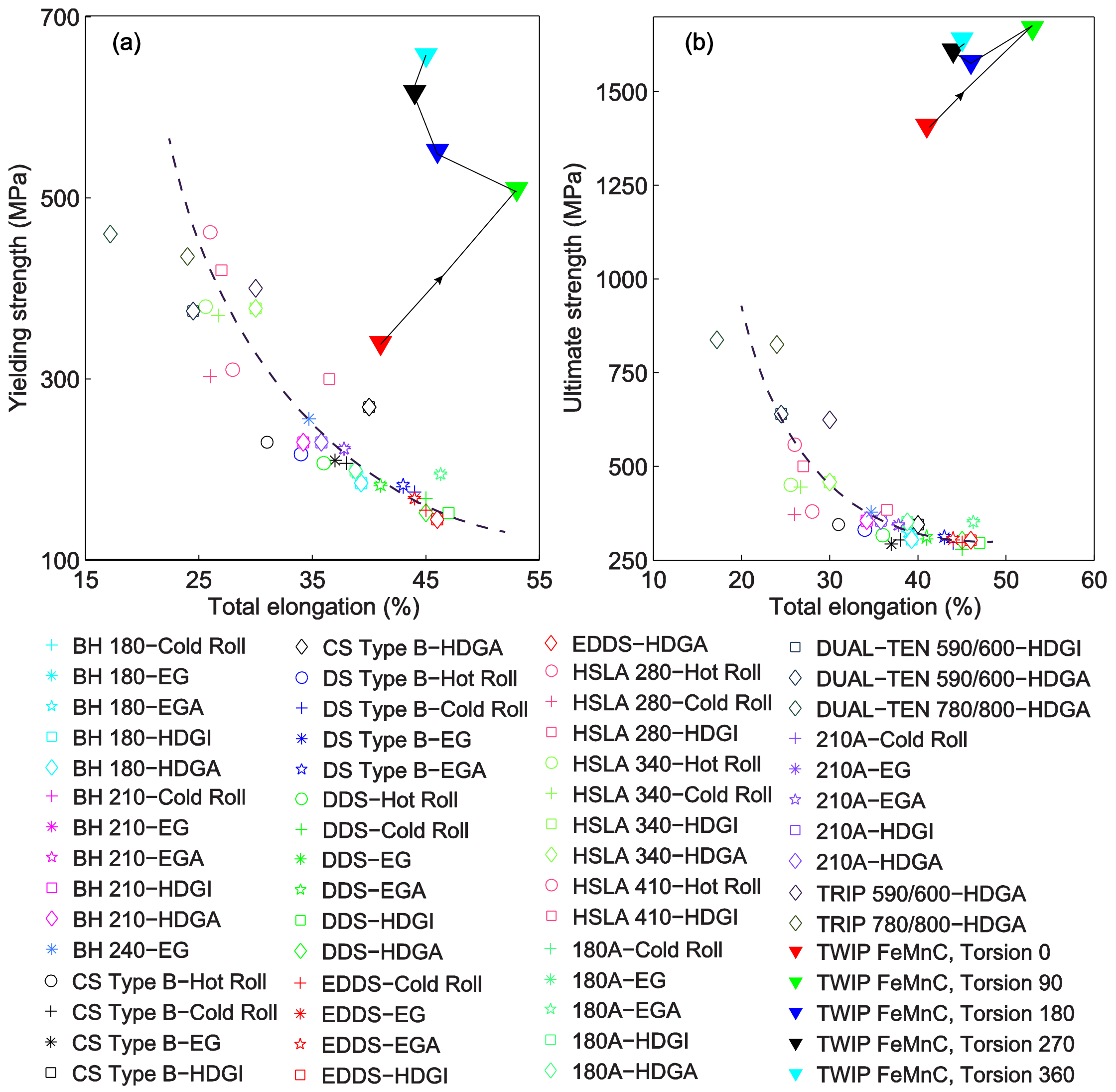

Supplementary Figure 1 | Strength versus total elongation for typical steels (data with filled circles come from this study. All other data come from US Steel Co.[1] The dashed line is for guidance to show the trade-off between yielding strength and ductility in most daily used steels. (**a**) The 0.2% yielding strength versus total elongation. (**b**) Ultimate tensile strength versus total elongation. The arrow in the solid lines points to the direction of increasing pre-torsion applied to the FeMnC TWIP steel. We note the significant increasing in yielding strength in FeMnC TWIP steel by pre-torsion.

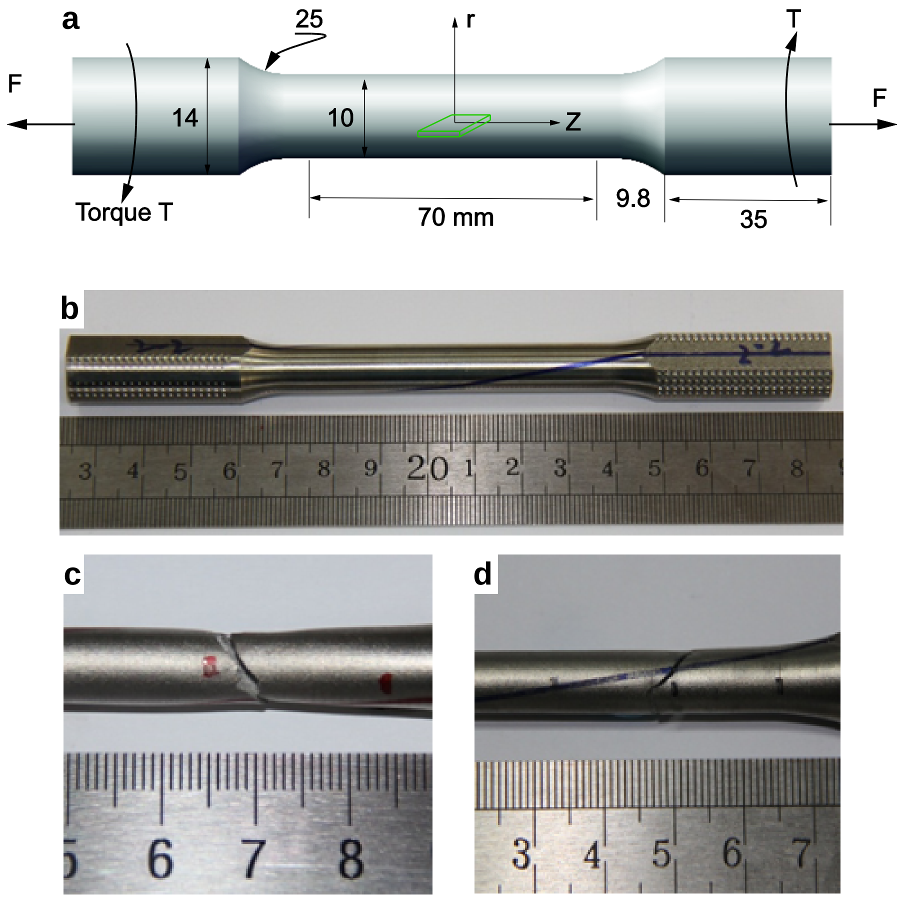

Supplementary Figure 2 | Sample dimensions and their failure mode. (**a**) Dimensions of the samples for torsion and tension tests. (**b**) Sample with 180° pre-torsion. Note that real rotation in the middle part of the sample with uniform diameter of 10mm is typically slightly smaller than the nominal torsion applied to the two ends of a sample. (**c**) Tensile failure mode of the sample without pre-torsion. (**d**) Tensile failure mode of the sample with pre-torsion. All samples failed by shearing with slight local necking.

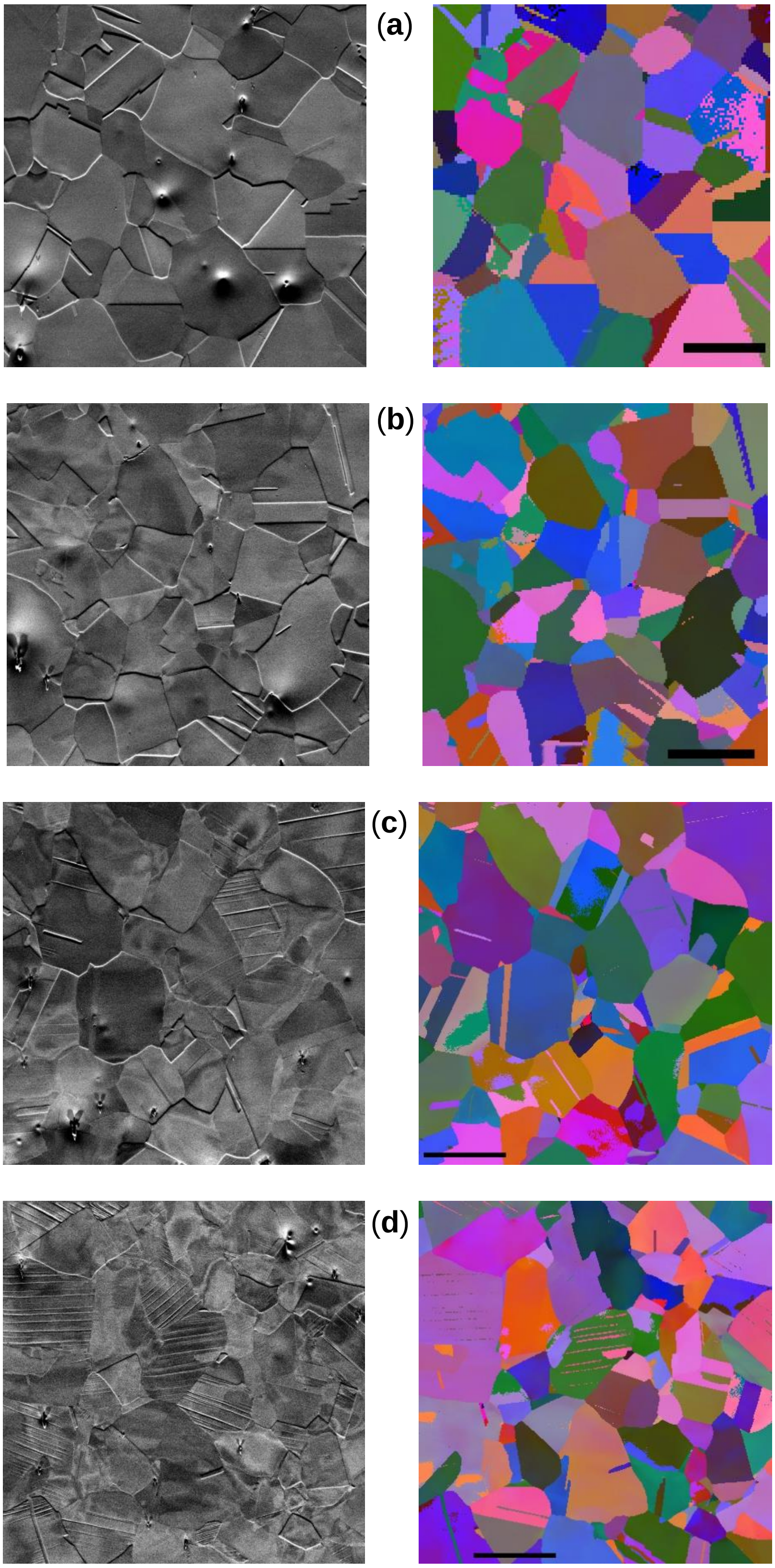

Supplementary Figure 3 | SEM images (left) and EBSD (right) images to reveal twins along radial positions in TWIP steel after 180° torsion: (**a**) r=0, (**b**) r=0.25R, (**c**) r=0.5R, and (**d**) r=R. Colors represent crystallographic orientations, by which twins in a grain can be distinguished. Twin gradient is seen: more twins at larger radius (scale bar 30 μm).

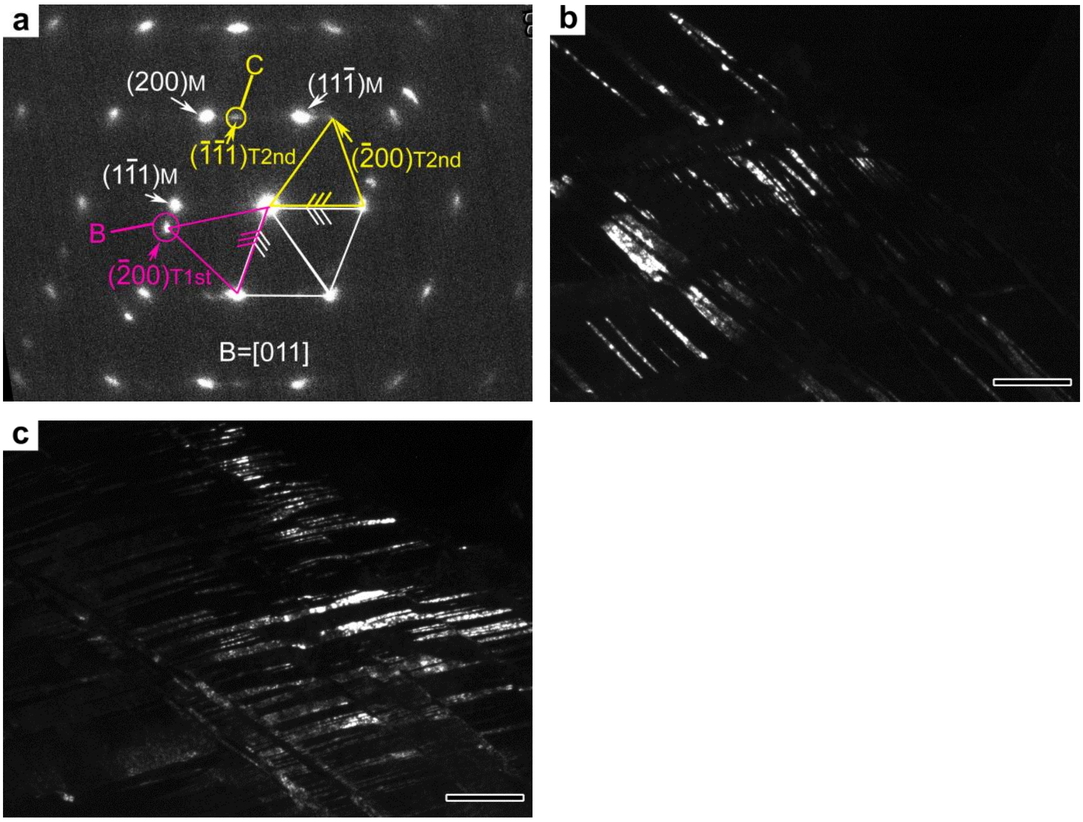

Supplementary Figure 4 | Detailed information about the twin structures shown in Fig. 3a. (**a**) The spots for primary and secondary twins in the SAED patterns with <011>-beam incident. The diffraction spots associated with secondary twins are weaker than those of primary twins, indicating that the secondary twins are finer than the primary ones. No individual spots for tertiary twins are observed in the SAED patterns since they have the same twining factors as the primary twins. (**b**) The dark field TEM image of the $(\bar{2}00)_{T1st}$ spot labeled by 'C' in (**a**), clearly shows primary and tertiary twins (scale bar 0.3 μm). (**c**) The dark field TEM image of the $(\bar{1}\bar{1}1)_{T2nd}$ spot indicated by 'D' in (**a**) (scale bar 0.3 μm).

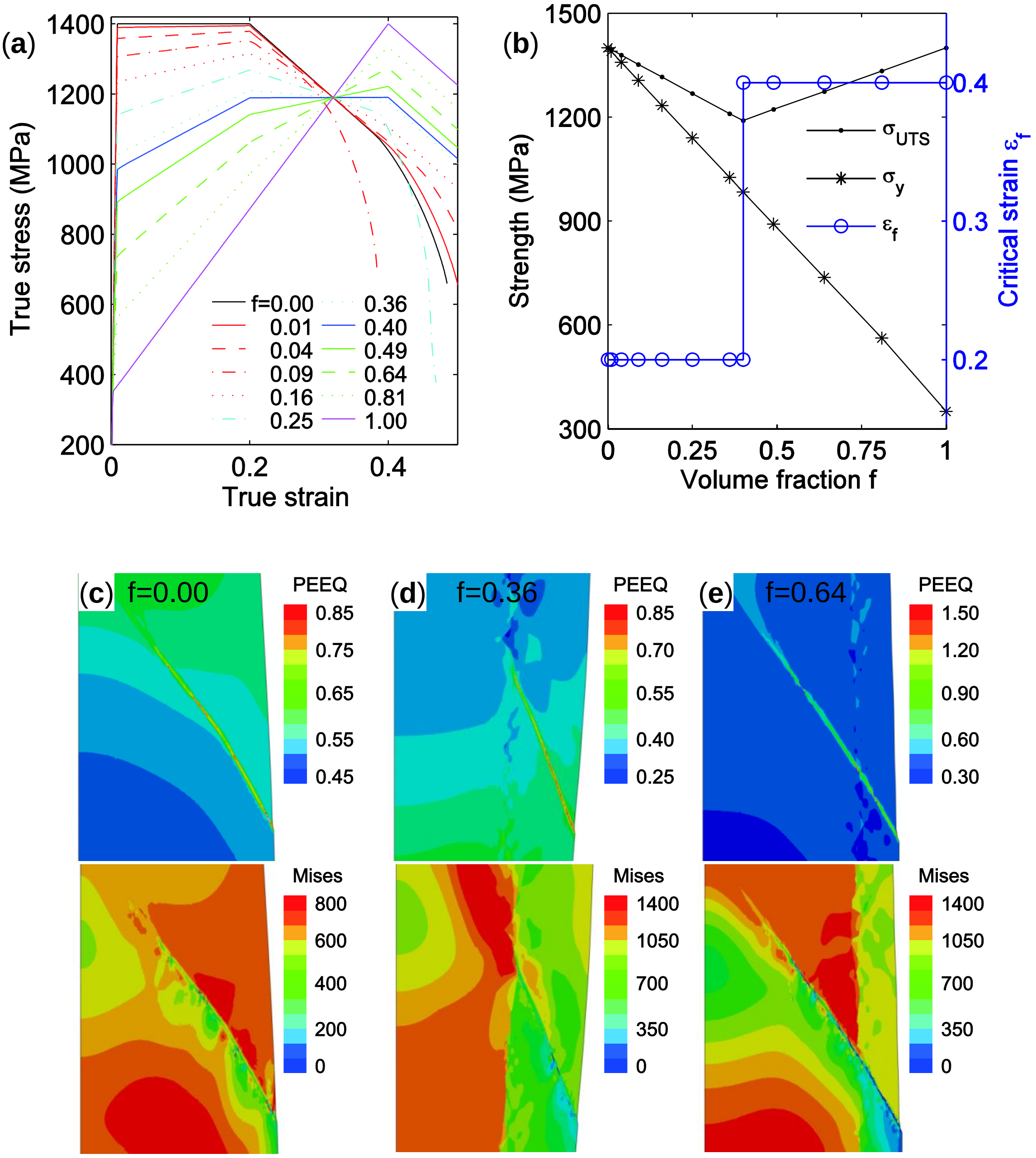

Supplementary Figure 5 | Critical hardening modulus for shear localization (Supplementary Note 1). (**a**) The stress-strain response of the sample with different *f*. (**b**) The yielding strength σ_{y}, the ultimate strength σ_{UTS}, and the macroscopic failure strain ε_{f} as functions of *f*. An abrupt transition in ε_{f} occurs at the point when $\bar{h}=0$. (**c**) to (**e**) Post-failure equivalent plastic strain (top) and stress contours (bottom), corresponding to *f*=0.0; *f*=0.36, and *f*=0.64, respectively. Deformation in the samples exhibits slight diffuse necking, followed by concentrated shear zones.

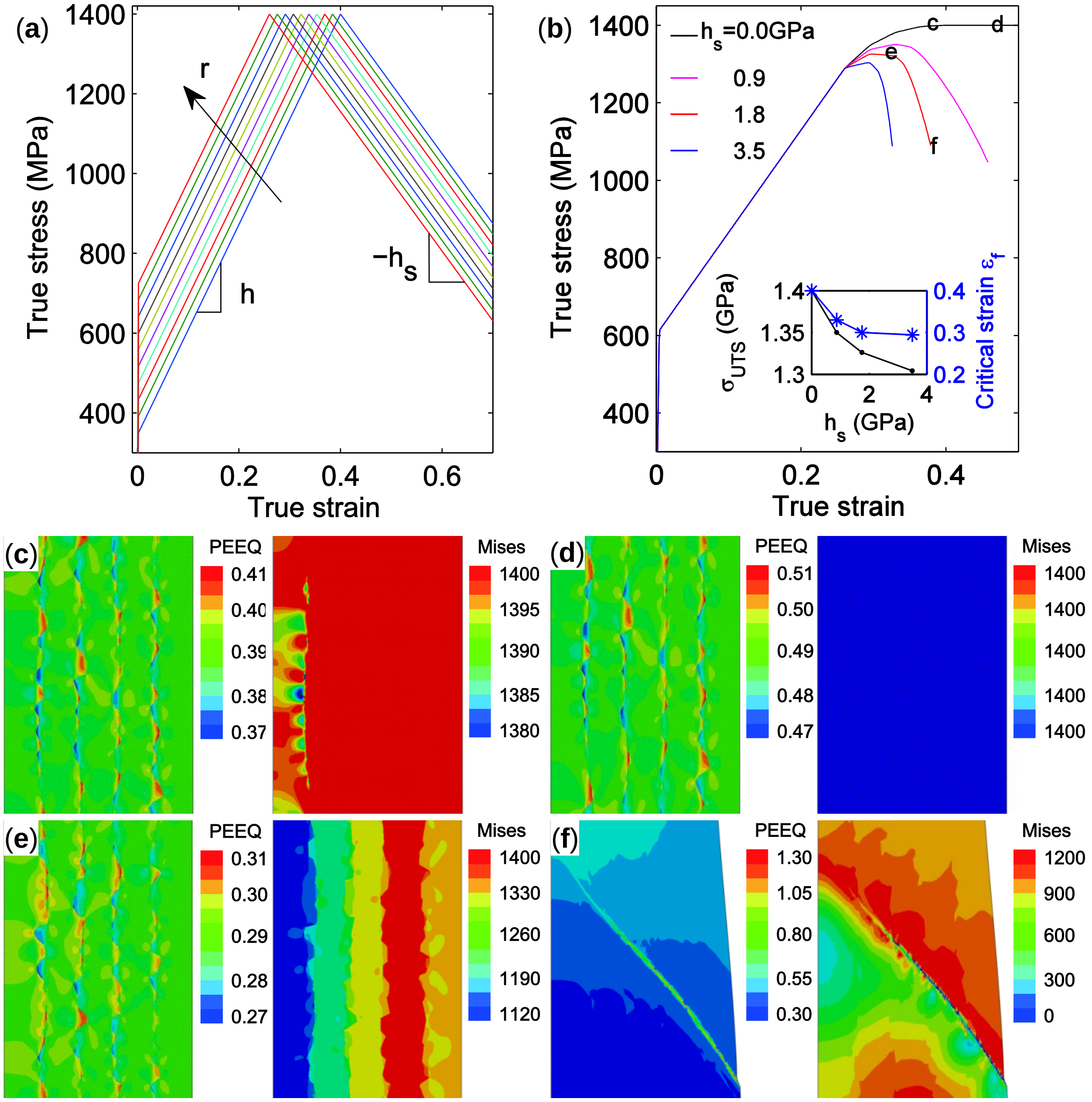

Supplementary Figure 6 | Influence of strain softening modulus on ductility (Supplementary Note 1). (**a**) Corresponding stress-strain curves for each region. (**b**) Macroscopic stress-strain behavior of the gradient twin sample with different softening moduli. Equivalent plastic strain and stress contours in the sample with different h_s at different strain levels: (**c**) $h_s = 0$, at 'c' in (b); (**d**) $h_s = 0$, at 'd' in (b); (**e**) $h_s = 1.8$ GPa and at 'e' in (b); (**f**) $h_s = 1.8$ GPa and at 'f' in (b).

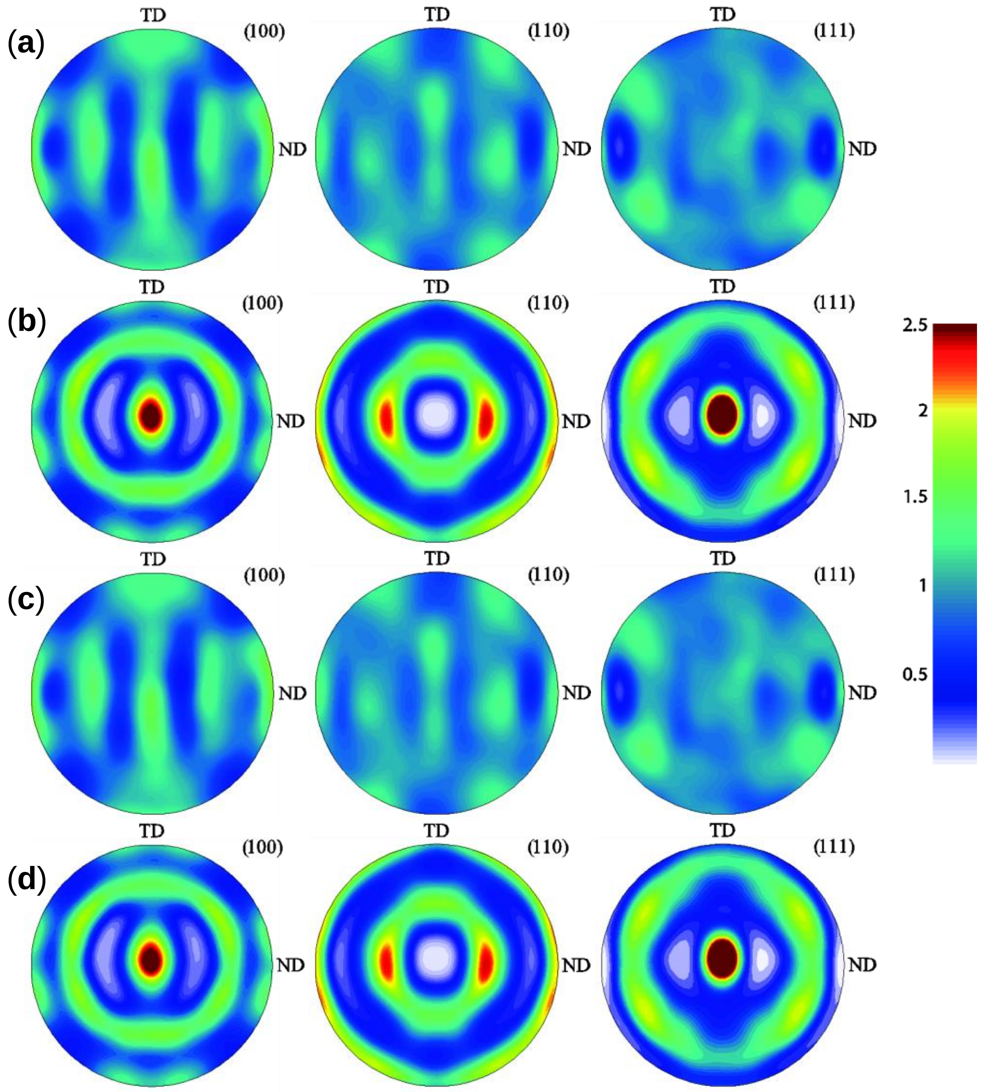

Supplementary Figure 7 | Calculated texture in TWIP steel samples at different stages of deformation. (**a**) Initial pole figures showing weak texture in three characteristic planes. (**b**) Pole figures at 40% tensile strain. (**c**) Pole figures after 180° pre-torsion. (**d**) Pole figures at 46% tensile strain in the 180° pre-torsioned sample. Note that the pore figures after pre-torsion are distinctly different from those after tension, suggesting the activation of different twinning systems by torsion and tension, which leads to non-simple additive effects of plastic strain by torsion and that by subsequent tension.

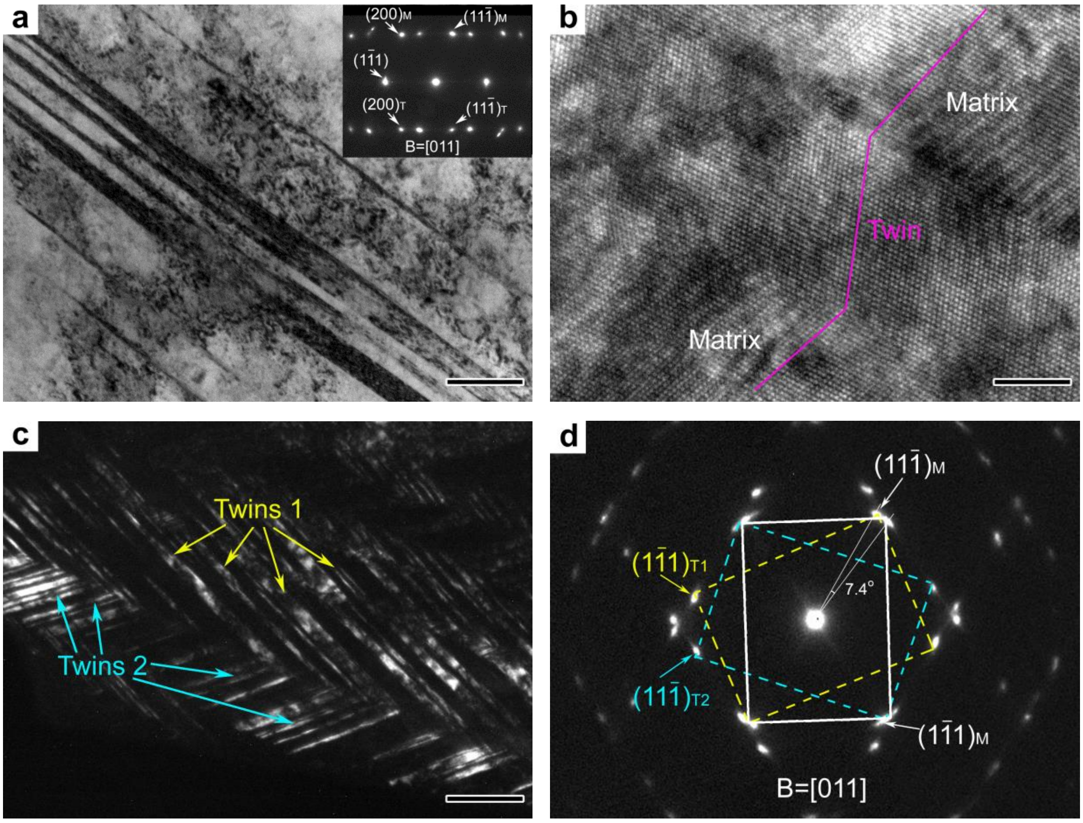

Supplementary Figure 8 | Microstructures in the core of the 180° pre-torsioned sample after tensile failure. (**a**) TEM image of the long parallel twins; inset is the corresponding SAED image showing typical twin patterns (scale bar 30 μm). (**b**) HRTEM image of a twin (scale bar 3 nm). (**c**) Dark-field TEM image of two sets of twins (scale bar 40 nm) and (**d**) the corresponding SAED images showing two sets of twin patterns. Twin sets 1 and 2 share the common matrix with a slight separation (7.4^{o}) of the originally common {111} diffraction spots. The slight separation of diffraction spots could be attributed to severe deformation which may make twin boundaries incoherent. Similar separation of diffraction spots were observed in Cu-30wt.%Zn alloys after high-pressure torsion[14].

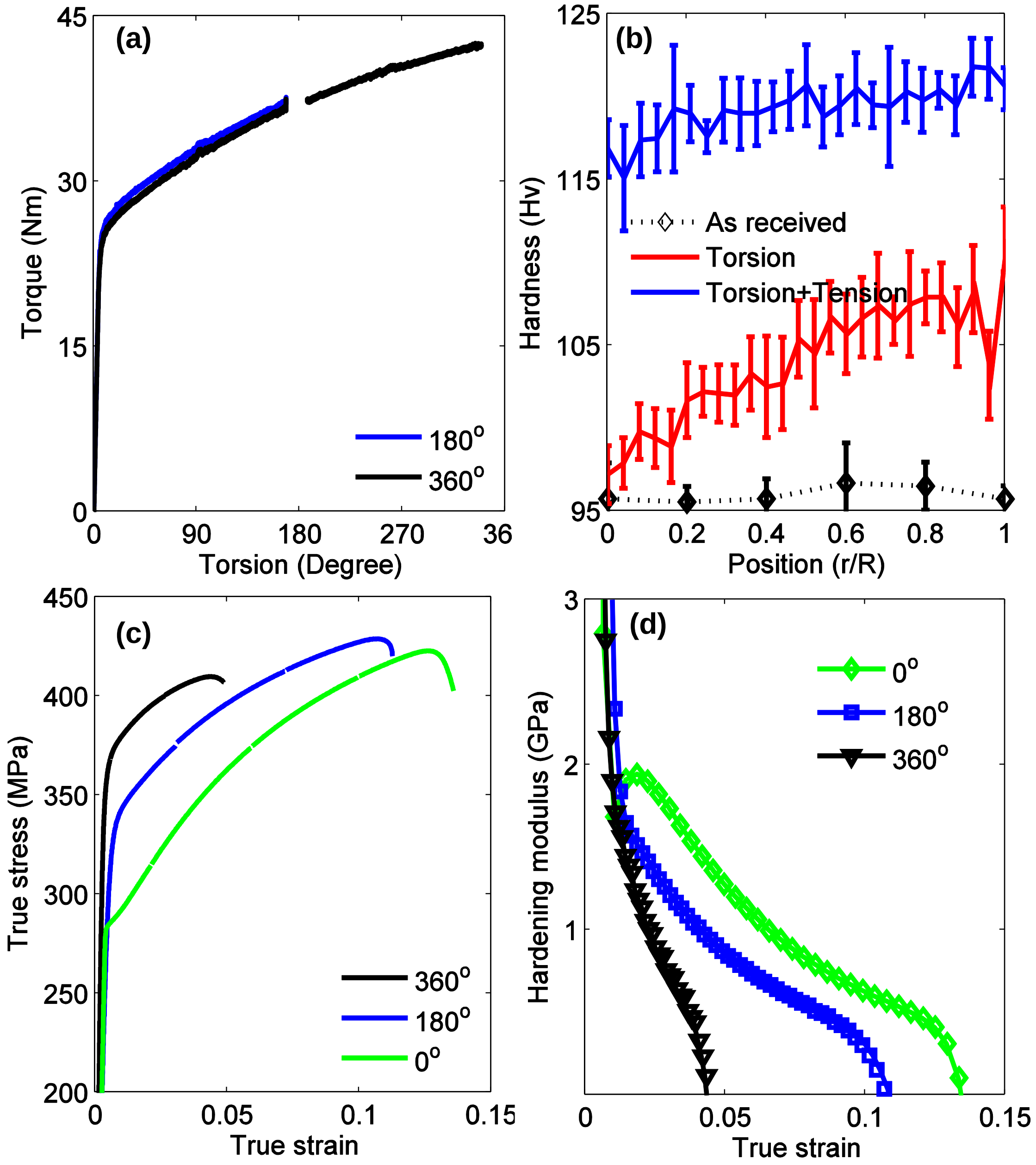

Supplementary Figure 9 | Mechanical behavior of pre-torsioned 6061-Aluminum bars. (**a**) Torque versus applied twist. (**b**) Evolution of hardness along the radial direction in the 180° pre-torsioned samples, and the 180° pre-torsioned samples after subsequent tension. (**c**) Stress-strain curves of samples subject to pre-torsion to different angles. While enhancement in their 0.2% yielding strength is clearly seen, the pre-torsion treatment also results in degradation in ductility due to the lack of a hierarchical nanotwin network. (**d**) The hardening modulus as a function of tensile strain on the pre-torsioned samples. The pre-torsion decreases the hardenability as strain increases, and causes reduction in tensile ductility.

Slip system		Twinning system	
ID	Slip plane/direction	ID	twin plane/direction
A1	$(111)[01\bar{1}]$	T1	$(111)[\bar{2}11]$
A2	$(111)[\bar{1}01]$	T2	$(111)[1\bar{2}1]$
A3	$(111)[1\bar{1}0]$	T3	$(111)[11\bar{2}]$
B1	$(\bar{1}\bar{1}1)[0\bar{1}\bar{1}]$	T4	$(\bar{1}\bar{1}1)[2\bar{1}1]$
B2	$(\bar{1}\bar{1}1)[101]$	T5	$(\bar{1}\bar{1}1)[\bar{1}21]$
B3	$(\bar{1}\bar{1}1)[\bar{1}10]$	T6	$(\bar{1}\bar{1}1)[\bar{1}\bar{1}\bar{2}]$
C1	$(\bar{1}11)[01\bar{1}]$	T7	$(\bar{1}11)[211]$
C2	$(\bar{1}11)[101]$	T8	$(\bar{1}11)[\bar{1}\bar{2}1]$
C3	$(\bar{1}11)[\bar{1}\bar{1}0]$	T9	$(\bar{1}11)[\bar{1}1\bar{2}]$
D1	$(1\bar{1}1)[0\bar{1}\bar{1}]$	T10	$(1\bar{1}1)[\bar{2}\bar{1}1]$
D2	$(1\bar{1}1)[\bar{1}01]$	T11	$(1\bar{1}1)[121]$
D3	$(1\bar{1}1)[110]$	T12	$(1\bar{1}1)[1\bar{1}\bar{2}]$

Supplementary Table 1 | The twelve slip systems and twelve twinning systems for face-centered cubic metals used in crystal plasticity simulations (Supplementary Note 2). Note that dislocations may slip in both directions, as long as the absolute resolved shear stress is greater than the critical value; however, twinning can only occur in one direction due to its pole nature.

Supplementary Note 1 | Finite element modeling on gradient structure

We performed a series of finite element (FEM) simulations to address why the combination of gradients and twin structures would be beneficial for both strength and tensile ductility. In particular, the simulations were aimed to investigate (a) the strengthening mechanism by gradient structure, (b) the importance of gradient structure for ductility improvement; and (c) the activation of different twinning systems during torsion and subsequent tension that leads to the formation of twin hierarchy and improved ductility. The plastic deformation is described by von Mises flow (also referred as J2-flow) rule. For the cylindrical sample with a hard shell and soft core with distinct stress-strain behavior (Fig. 4a),material in the hard shell has high yield strength and starts to soften at $\varepsilon_{\mathrm{f}}^{\mathrm{hard}}$. In contrast, material in the core hardens until 40% strain, beyond which softening begins. In their respective softening regions, the same softening modulus $h_{\mathrm{s}} = 1.75\,\mathrm{GPa}$ is used for the core and the shell. The representative two-dimensional polycrystalline microstructure used in the calculations is composed of uniform voronoi grains.[2] Each grain, depending on its radial location, is embedded with an array of parallel twin boundaries. Regions with higher twin densities also have higher yielding strength. In our 2D axi-symmetric samples, since the isotropic plasticity model is sufficient to illustrate the essential physics, grains in each strength regime are not differentiable. The commercial software Abaqus 6.11 standard[3] is used to simulate plasticity in 2D axi-symmetric samples. The 2D sample contains 824 grains and is meshed with 332787 CAX3 and CAX4 axi-symmetrical elements.

To explore the localization condition in an axi-symmetric, gradient twin sample subjected to tension, we simulated the response of a cylindrical sample with fixed mechanical properties in the hard shell and soft core regions, while changing the volume fraction *f* of the soft core. We adopted the same structure shown in Fig. 4b. Material properties in the soft core is kept the same but the softening point of the hard core is now fixed at $\varepsilon_{\mathrm{f}}^{\mathrm{hard}} = 0.2$. Supplementary Figure 5a shows the stress-strain response of the sample with different values of the volume fraction *f*. A summary of the yielding strength

σ_y, the ultimate strength σ_{UTS}, as well as the critical failure strain ε_f is given in Supplementary Fig. 5b. The yielding strength increases linearly with increasing volume fraction *f* of the soft core, suggesting a simple law of mixture for the yielding strength of the gradient twin structure. Meanwhile, we observe an abrupt transition in ε_f, which occurs at the point when $\bar{h} = f\,h - (1-f)h_s$ is zero. This critical hardening modulus for localization is consistent with the prediction by Rice[4] for plastic materials with smooth yield loci, but differs from the Considère criterion[5] for instability ($\partial\sigma / \partial\varepsilon - \sigma = 0$ where σ and ε are the uniaxial stress and strain, respectively) based on the maximum load capacity of the sample as well as the generalized Considère criterion incorporating strain-rate sensitivity[6]. The post-failure equivalent plastic strain contours and equivalent stress contours are shown in supplementary Figs. 5c to e, corresponding to volume fractions of *f*=0.0; *f*=0.36, and *f*=0.64, respectively. Deformation in all samples exhibited slight diffuse necking followed by concentrated shear zones.

For a pre-torsioned sample with linear strength gradient (which is supported by the almost linear hardening curve of TWIP steel in the absence of pre-torsion, see Fig. 1c), we divide the sample evenly into ten regions along the radial direction. After taking their deformation history into account, we assume each region has a stress-strain response shown in supplementary Fig. 6a. The macroscopic stress-strain behavior of the gradient twin sample with different softening moduli is shown in supplementary Fig. 6b. It again shows that the gradient twin structure could increase the yielding strength and the ductility of the sample, and low strain softening could improve both the strength and ductility of the sample as well. The equivalent plastic strain and stress contours at the total strain of 0.4 for $h_s = 0$ are shown in supplementary Fig. 6c to d, respectively, with no sign of localization. When $h_s = 1.8$ GPa, strain localization is seen after a peak stress from the equivalent plastic strain and stress contours in supplementary Figures 6e and f.

The FEM simulations shown in Fig. 4 and supplementary Figs. 5 and 6 clearly indicate that the gradient twin structure could (a) profoundly increase the yielding strength

beyond that of the soft-core, following a simple law of mixture $\bar{\sigma}_{y} = \left(\int_{S} \sigma_{y} dA\right) A^{-1}$; (b) the gradient could also improve the ductility in comparison with that of the hard shell, even reaching the same failure strain as that of the soft-core; (c) the localization of the axi-symmetric, gradient twin sample in tension would occur when the composite hardening modulus $\bar{h}$ =0. It is important to note that we assumed that the pre-straining by torsion degrades the ductility of the sample (the hard-core has higher yield strength but lower critical strain where softening starts) in calculations shown in supplementary Fig. 6. This assumption could be too conservative as deformation twinning by torsion in TWIP steel may not necessarily degrade the ductility. As seen from the TEM/SEM pictures (Fig. 2), after torsion the deformation twins in individual grains are very clean and parallel. During subsequent tension, such preferential twinning systems may give their way to other twinning systems that can be further activated.

Supplementary Note 2 | Crystal plasticity modeling on active slip/twinning systems

We employ the classical framework of rate-independent single-crystal plasticity which considers both dislocation slip and deformation twinning in face-centred cubic metals.[7] For self-consistency, we include here some of the key ingredients in the model. In such framework, the deformation gradient $\mathbf{F}$ is decomposed into elastic ($\mathbf{F}^{\mathrm{e}}$) and plastic ($\mathbf{F}^{\mathrm{p}}$) parts as

$$\mathbf{F} = \mathbf{F}^{\mathrm{e}}\mathbf{F}^{\mathrm{p}}. \tag{1}$$

Plastic flow takes place through both dislocation slip and deformation twinning in prescribed crystallographic slip/twinning systems. Using integer i to label crystal slip systems and integer α to label crystal twin systems, respectively, each slip system is defined by a slip/twinning direction $\mathbf{m}_0$ and a slip/twinning-plane normal $\mathbf{n}_0$ (the complete list of all dislocation slip and twinning systems is given in Supplementary Table 1). The Green elastic strain measure $\mathbf{E}^{\mathrm{e}}$ and the symmetric second Piola-Kirchhoff stress tensor $\mathbf{T}$ are defined respectively as

$$\mathbf{E}^{\mathrm{e}} \equiv (1/2)(\mathbf{F}^{\mathrm{eT}}\mathbf{F}^{\mathrm{e}} - \mathbf{1}) \text{ and } \mathbf{T} \equiv \mathbf{C}\mathbf{E}^{\mathrm{e}}, \tag{2}$$

where the superscript 'T' stands for the transpose of a tensor and '$\mathbf{C}$' is the fourth order tensor of elastic moduli. The resolved shear stress τ on slip/twinning system ($\mathbf{m}_0, \mathbf{n}_0$) is calculated as

$$\tau = (\mathbf{F}^{\mathrm{eT}}\mathbf{F}^{\mathrm{e}}\mathbf{T}) \cdot (\mathbf{m}_0 \otimes \mathbf{n}_0). \tag{3}$$

The evolution of plastic deformation gradient, on multiple i-/α-th slip/twin systems, is given as

$$\mathbf{F}^{\mathrm{p}} = \mathbf{1} + \sum_i \Delta\gamma^i \mathbf{S}_0^i + \sum_\alpha \Delta\gamma^\alpha \mathbf{S}_0^\alpha, \mathbf{S}_0^i \equiv \mathbf{m}_0^i \otimes \mathbf{n}_0^i \text{ and } \mathbf{S}_0^\alpha \equiv \mathbf{m}_0^\alpha \otimes \mathbf{n}_0^\alpha \tag{4}$$

where $\mathbf{S}_0^i$ is the Schmid tensor for the i-th slip system, and $\mathbf{S}_0^\alpha$ is the Schmid tensor for the α-th twin system; $\Delta\gamma$ is the incremental shear strain due to slip/twinning, which is determined by using the consistency condition in rate-independent plasticity scheme given the resolved shear stress τ and resistance $\mathbf{S}$ on the slip/twinning system. Twinning is considered as pseudo-slip, and the lattice in the region of the crystal that has gone

through twinning rotates with respect to a characteristic axis[8-10]. For TWIP steel, its twin elements $\{K_1, K_2, \eta_1, \eta_2\}$ are given as[11]

$$K_1 = (111),\ \eta_1 = [11\bar{2}],\ K_2 = (11\bar{1}),\ \eta_2 = [112]. \quad (5)$$

With $\mathbf{p}$ as a unit vector in the $\eta_1 = [11\bar{2}]$ direction, and $\mathbf{q}$ as the unit normal to the $K_1 = (111)$ plane, the rotations which carry the matrix lattice to twinned lattice and the inverse (or detwinning) are given by, respectively,

$$\mathbf{R}^{\mathrm{tw}} = 2\mathbf{q} \otimes \mathbf{q} - 1 \text{ and } \mathbf{R}^{\mathrm{dw}} = 2\mathbf{p} \otimes \mathbf{p} - \mathbf{1}. \quad (6)$$

The twelve slip systems in FCC crystals are $\{111\} < 110 >$, and the twelve twin systems in FCC crystal are $\{111\} < 11\bar{2} >$. The initial resistance to slip is taken to be 121MPa and that to twin 70MPa. These numbers reflect the fact that the nucleation of leading partial dislocations for twins is easier than the generation of complete dislocations.[12] Initially a random texture was assigned to the grains. The reader is referred to Ref. 13 for more detailed information about the application of the crystal plasticity model to plastic deformation in TWIP steel. Two different boundary value problems, similar to the experimental set up, were considered. For the first case, we apply simple tension to the bar until about 40% strain. In another independent simulation, we twist the bar first, and then apply uniaxial tension. During each stage of deformation, we calculate the equivalent plastic strain rates contributed respectively by individual cryptographic slip systems and twinning systems in an element as follows[13]:

$$\dot{\bar{\gamma}}_{\mathrm{sl}} = \sqrt{2/3\left(\sum_i \dot{\gamma}^i_{\mathrm{sl}} \mathbf{S}^i_0\right) : \left(\sum_i \dot{\gamma}^i_{\mathrm{sl}} \mathbf{S}^i_0\right)},\quad \Delta\gamma_{\mathrm{sl}} = \int \dot{\bar{\gamma}}_{\mathrm{sl}}\, dt, \quad (7)$$

where i covers all the slip systems, and

$$\dot{\bar{\gamma}}_{\mathrm{tw}} = \sqrt{2/3\left(\sum_\alpha \dot{\gamma}^\alpha_{\mathrm{tw}} \mathbf{S}^\alpha_0\right) : \left(\sum_\alpha \dot{\gamma}^\alpha_{\mathrm{tw}} \mathbf{S}^\alpha_0\right)},\quad \Delta\gamma_{\mathrm{tw}} = \int \dot{\bar{\gamma}}_{\mathrm{tw}}\, dt, \quad (8)$$

where α spans over all the twinning systems. In the above equations, $\dot{\gamma}^i_{\mathrm{sl}}$ and $\dot{\gamma}^\alpha_{\mathrm{tw}}$ are the shear strain rates for the i-th slip system and the α-th twin system, respectively. With the plastic shear strains $\Delta\gamma_{\mathrm{sl}}$ and $\Delta\gamma_{\mathrm{tw}}$ obtained for each slip/twinning systems,

we then calculated the average plastic strain contributed by each slip/twinning systems over all elements in a grain. Abaqus 6.11 Explicit[3] is used for the crystal plasticity simulations and the constitutive model is implemented by using the user subroutine function. The 3D microstructure (Fig. 5a) used for our crystal plasticity simulations contains 300 grains and 58806 C3D4 elements. We chose two grains residing in the surface of the bar for demonstration, with Euler angles (ϕ, θ, ω) of those two grains give as (G1: (17.5^o,178°,172°)) and (G2: (52.5^o,53°,137°)).

Supplementary References

1. US Steel Cooperation. A comparison chart of mechanical properties pertaining to every steel available for use in automotive applications today. http://www.ussteel.com/uss/portal/home/markets/automotive (2013).
2. Quey, R., Dawson, P.R. & Barbe, F. Large-scale 3D random polycrystals for the finite element method: Generation, meshing and remeshing. *Comp. Meth. App. Mech. Eng.* **200,** 1729–1745 (2011).
3. Abaqus FEA, D S Simulia. © Dassault Systèmes, 2007.
4. Rice, J. R. The localization of plastic deformation, in Theoretical and Applied Mechanics (Proc. of the 14th International Congress on “Theoretical and Applied Mechanics”, Delft, 1976. Ed. Koiter, W.T., Vol. 1, North-Holland Publishing Co. 207-220 (1976).
5. Considère, A. Mémoire sur l'emploi du fer et de l'acier dans les constructions. Ann. *des Ponts Chaussées* **9,** 574–775 (1885).
6. Hart, E.W. Theory of the tensile test. *Acta Metall*. **15,** 351–355 (1967).
7. Staroselsky, A., Anand, L. Inelastic deformation of polycrystalline face centered cubic materials by slip and twinning. *J. Mech. Phys. Solids* **46,** 671–696 (1998).
8. Kelly, A.A. & Knowles, K.M. *Crystallography and Crystal Defects* (John Wiley & Sons, London, 2012).
9. Pitteri, M. On v+1-lattices. *J. Elast.* **15,** 3-25 (1985).
10. Pitteri, M. On type-2 twins in crystals. *Int. J. Plast*. **2,** 99-106 (1986).

11. Christian, J.W. & Mahajan, S. Deformation twinning. *Prog. Mater. Sci*. **39,** 1-157 (1995).
12. Asaro, R.J., Krysl, P. & Kad, B. Deformation mechanism transitions in nanoscale fcc metals. *Philos. Mag. Lett*. **83,** 733-743 (2003).
13. Li, Y., Zhu, L., Liu, Y., Wei, Y., Wu, Y., Tang, D. & Mi, Z. On the strain hardening and texture evolution in high manganese steels: experiments and numerical investigation. J. Mech. Phys. Solids 61, 2588-2604 (2013).
14. Wang, Y.B., et al. The role of stacking faults and twin boundaries in grain refinement of a Cu–Zn alloy processed by high-pressure torsion. *Mater. Sci. Eng. A* **527,** 4959–4966 (2010).
